# Complete Genome Sequence of a Colombian Zika Virus Strain Obtained from BALB/c Mouse Brain after Intraperitoneal Inoculation

**DOI:** 10.1128/MRA.01719-18

**Published:** 2019-11-14

**Authors:** Katherine Laiton-Donato, Diego A. Álvarez-Díaz, Aura Caterine Rengifo, Orlando Torres-Fernández, José A. Usme-Ciro, Jorge Alonso Rivera, Gerardo Santamaría, Julián Naizaque, Jeison Monroy-Gómez, Ladys Sarmiento, María Luz Gunturiz, Alejandra Muñoz, Ricardo Vanegas, Angélica Rico, Lissethe Pardo, Dioselina Peláez-Carvajal

**Affiliations:** a Grupo de Virología, Dirección de Redes en Salud Pública, Instituto Nacional de Salud, Bogotá, DC, Colombia; b Grupo de Morfología Celular, Dirección de Investigación en Salud Pública, Instituto Nacional de Salud, Bogotá, DC, Colombia; c Grupo Animales de Laboratorio, Dirección de Producción, Instituto Nacional de Salud, Bogotá, DC, Colombia; d Escuela Colombiana de Rehabilitación, Bogotá, DC, Colombia; e Equipo Banco de Proyectos, Dirección de Investigación en Salud Pública, Bogotá, DC, Colombia; f Centro de Investigación en Salud para el Trópico–CIST, Universidad Cooperativa de Colombia, Santa Marta, Colombia; KU Leuven

## Abstract

A Zika virus (ZIKV) strain was isolated from an acute febrile patient during the Zika epidemics in Colombia. The strain was intraperitoneally inoculated into BALB/c mice, and 7 days postinoculation, neurological manifestations and ZIKV infection in the brain were demonstrated. The reported genome sequence is highly related to strains circulating in the Americas.

## ANNOUNCEMENT

Zika virus (ZIKV) is the etiological agent of an emerging viral disease with serious public health impact (1, 2). It belongs to the family Flaviviridae, genus Flavivirus, and possesses a positive-sense, single-stranded RNA genome of approximately 10.8 kb. From August 2015 to November 2016, Colombia reported 105,000 cases of ZIKV infection, and 147 cases of microcephaly were confirmed by laboratory diagnosis (3). Different factors have been associated with the development of ZIKV neuropathogenesis, including the selective infection and damage of neural progenitor cells (4) and antibody cross-reactivity leading to a demyelinating subtype of Guillain Barré syndrome (GBS) (5, 6). Experimental infection in an animal model could be valuable for identifying specific mutations allowing neuroinvasiveness and neurovirulence (7). Thus, BALB/c mice were infected with ZIKV as a means to understand the neuropathogenesis of Zika.

A serum sample from a pregnant woman diagnosed according to the WHO classification of diseases (International Classification of Diseases, 10th Revision) with mosquito-borne viral fever, unspecified (A92.9) (https://icd.who.int/browse10/2016/en#/A92-A99), was collected in the city of Villavicencio (Meta, Colombia) on 17 January 2016 and submitted to the Instituto Nacional de Salud (INS)–Colombia for laboratory diagnosis as part of the national public health surveillance system. ZIKV infection was confirmed by real-time reverse transcription-PCR (RT-PCR) Trioplex assay (Chikungunya virus [CHIKV]-dengue virus [DENV]-ZIKV) (8). Serum was diluted 1:100 in minimal essential medium (MEM), and 200 μl of the dilution was inoculated in Vero cells. Cytopathic effect was observed 6 days after inoculation, and ZIKV was confirmed in cell supernatants by Trioplex assay.

Five newborn mice were inoculated with ZIKV and mock infected with their respective controls by the intracerebral pathway. In this assay, coinfection of ZIKV and CHIKV was detected in the brains using the Trioplex assay, although no CHIKV was detected in the cell culture inoculated with the isolate. The initial inoculum contained CHIKV at a level below the detection limit of the assay (coinfections are frequently encountered in our region), and the CHIKV titers increased in the mouse brain due to the strong capacity of CHIKV to infect neurons (9, 10).

To obtain a pure ZIKV strain without coinfections, a plaque-to-plaque transfer assay was performed. Seven lysis plaques (clones 1 to 7) were taken randomly with a syringe needle and cultured in Vero cells in independent assays. Five days postinoculation, 200 μl of the supernatants was reinoculated into a fresh monolayer, and 7 successive passages of each clone were done with the same strategy. This was done to rule out the growth of other possible viruses in these assays. Six of the clones (clones 2 to 7) were positive only for ZIKV, while clone 1 showed coinfection with CHIKV.

The ZIKV clone 7 isolate was named Zika_virus_459148_Meta_Colombia_2016. The third passage of this isolate was titrated (1.25 × 10^7^ PFU/ml); 40 μl was inoculated intraperitoneally into 10 newborn mice on postnatal day 1, and control animals were inoculated with culture supernatants of uninfected Vero cells.

The presence of neurological manifestations such as hypersensitivity to touch, action tremor, and gait instability 7 days postinfection suggested that the strain was able to cross the developing blood-brain barrier. Fresh whole brains were collected by manual dissection; one cerebral hemisphere was immersed in RNAlater and stored at −70°C for molecular assays, and the other hemisphere was fixed in 4% buffered paraformaldehyde (PFA) solution for immunohistochemistry assays. The presence and purity of ZIKV was confirmed by immunoassays against ZIKV (anti-ZIKV pAb, lot 6 1576; donated by the CDC) and by conventional and real-time RT-PCR. Animal procedures were performed with the approval of the INS Animal Care and Use Committee (code 13-2016).

RNA purification from brain tissues was performed using the RNeasy lipid tissue minikit (Qiagen, Hilden, Germany), followed by cDNA synthesis with SuperScript IV reverse transcriptase (Thermo Fisher Scientific, Waltham, MA, USA) and random hexamers (Promega, Dübendorf, Switzerland). One RNA sample from the brain was taken for ZIKV sequencing. The library was prepared with the Nextera XT DNA library prep kit (Illumina, San Diego, CA, USA). Sequencing was performed with the MiSeq reagent kit version 2 (Illumina) on a MiSeq instrument (2 × 300 bp). Reads were demultiplexed by barcode, and a total of 423,712 reads (paired ends) were obtained and quality filtered with FastQC using a *Q*-score threshold of 30. A total of 5,031 reads mapped directly to a reference genome (GenBank accession number NC_035889) with Bowtie2 version 2.0.0-beta7, with nucleotide similarity coverages of 99.6% and 99.57%.

The polyprotein annotation was obtained through alignment with the Brazilian reference sequence (GenBank accession number YP_009428568 ). Ten nonsynonymous substitutions, namely D107E (capsid protein C), H691Y and E940K (membrane glycoprotein M), A1027T, K1059E, R1118W, and V1143M (envelope protein E), and I2509T, T3213I, and T3353A (nonstructural protein NS1), were observed in the polyprotein region (10,269 bp) when the sequence of Zika_virus_459148_Meta_Colombia_2016 was compared to the Brazilian reference sequence.

In summary, we report here the sequence of a ZIKV strain that is closely related to circulating strains in the Americas and able to infect brain tissue and produce neurological signs in a model of developing murine brain.

### Data availability.

The ZIKV genome sequence obtained from brains of infected BALB/c mice has been deposited in GenBank under the nucleotide accession number MH544701 and the Sequence Read Archive (SRA) accession number SRX4553103.
